# Management of imported malaria cases and healthcare institutions in central China, 2012–2017: application of decision tree analysis

**DOI:** 10.1186/s12936-019-3065-7

**Published:** 2019-12-18

**Authors:** Xi-Liang Wang, Jie-Bin Cao, Dan-Dan Li, Dong-Xiao Guo, Cheng-Da Zhang, Xiao Wang, Dan-Kang Li, Qing-Lin Zhao, Xiao-Wen Huang, Wei-Dong Zhang

**Affiliations:** 1 Department of Epidemiology, School of Public Health, Zhengzhou University, Zhengzhou, 450001 Henan People’s Republic of China; 2 The Centre for Disease Control and Prevention of Erqi District, Zhengzhou, 450001 Henan People’s Republic of China; 3 The First Affiliated Hospital of Zhengzhou University, Zhengzhou, 450001 Henan People’s Republic of China; 4 Department of International Medicine, Beaumont Health System, Royal Oak, MI 48073 USA

**Keywords:** Imported malaria, Henan, Healthcare institutions, Diagnosis, Complications, Management

## Abstract

**Background:**

Imported malaria has been an important challenge for China. Fatality rates from malaria increased in China, particularly in Henan Province, primarily due to malpractice and misdiagnoses in healthcare institutions, and the level of imported malaria. This study aims to investigate the relationship between the state of diagnosis and subsequent complications among imported malaria cases at healthcare institutions, based on malaria surveillance data in Henan Province from 2012 to 2017.

**Methods:**

A retrospective descriptive analysis was performed using data from the Centre for Disease Control and Prevention, Zhengzhou City, the capital of Henan Province. A decision tree method was exploited to provide valuable insight into the correlation between imported malaria cases and healthcare institutions.

**Results:**

From 2012 to 2017, there were 371 imported malaria cases, mostly in males aged between 20 and 50 years, including 319 *Plasmodium falciparum* cases. First visits of 32.3%, 19.9% and 15.9% malaria cases for treatment were to provincial, municipal and county healthcare institutions, respectively. The time interval between onset and initial diagnosis of 284 cases (76.5%) and the time interval between initial diagnosis and final diagnosis of 197 cases (53.1%) was no more than 72 h. An apparent trend was found that there were notably fewer patients misdiagnosed at first visit to healthcare institutions of a higher administrative level; 12.5% of cases were misdiagnosed in provincial healthcare institutions compared to 98.2% in private clinics, leading to fewer complications at healthcare institutions of higher administrative level due to correct initial diagnosis. In the tree model, the rank of healthcare facilities for initial diagnosis, and number of days between onset and initial diagnosis, made a major contribution to the classification of initial diagnosis, which subsequently became the most significant factor influencing complications developed in the second tree model. The classification accuracy were 82.2 and 74.1%, respectively for the tree models of initial diagnosis and complications developed.

**Conclusion:**

Inadequate seeking medical care by imported malaria patients, and insufficient capacity to diagnose malaria by healthcare institutions of lower administrative level were identified as major factors influencing complications of imported malaria cases in Henan Province. The lack of connection between uncommon imported malaria cases and superior medical resources was found to be the crucial challenge. A web-based system combined with WeChat to target imported malaria cases was proposed to cope with the challenge.

## Background

In spite of unprecedented global success in fighting malaria during first decade of the twenty-first century, performance has begun to plateau, chiefly resulting from limited funds, regional conflicts, abnormal climate, resistance of parasites to anti-malarials and resistance of mosquitoes to insecticides [1–4]. In addition, global economic integration and consequential increase in mobility of populations has not confined malaria to former geographic distribution, making control efforts complicated and progress towards elimination slow down [5–8].

China is a model of countries progressing to malaria elimination. As China forms close connections with many countries, just like its partners all over the world, malaria is undergoing profound changes. From 2006 to 2016, malaria cases in China decreased from 64,178 to 3142. Under the National Malaria Elimination Action Plan (2010–2020) (NMEAP) by the former Ministry of Health [9], Chinese indigenous cases sharply declined by 99.9% from 4262 cases in 2010 to three cases in 2016. China has entered the post-elimination era of malaria since, for the first time in 2017, there was no indigenous case reported [10]. Conversely, imported malaria cases increased and became a main challenge to malaria elimination in China [11].

*Plasmodium falciparum*, mainly imported from other countries, has taken the place of *Plasmodium vivax* that was historically the predominant species in China [10, 12]. Henan Province achieved the goal of zero indigenous malaria cases since 2012, 2 years after the initiation of NMEAP, whereas imported malaria cases, consisting chiefly of *P. falciparum*, have increased and become the foremost challenge to malaria elimination [13, 14].

Malaria cases in Henan Province after 2012 were mainly introduced from African countries where *P. falciparum* was the most prevalent malarial species [15]. Labourers infected by malaria parasites returning from African countries to their hometowns, primarily scattered in vast rural areas, bring randomized and sporadic cases to medical personnel unfamiliar with rare malaria cases or with the potential for fatality in cases of falciparum malaria [16]. After 2010, at the start of the NMEAP, the fatality rate from malaria in China increased and more than four-fifths of deaths had severe complications, such as brain lesions, shock, severe liver/kidney lesions, haemolysis [17]. Henan Province ranked first in the number of malaria deaths in the whole of China. All the 14 malaria deaths between 2010 and 2015 in Henan Province were falciparum malaria cases imported from Africa; 12 of them had attended more than one medical institution before an authoritative and correct diagnosis was made; the 6.5 days spent may undermine the efforts based on Chinese 1-3-7 strategy [18, 19].

On the one hand, the notable mortality from imported malaria in Henan Province results from low awareness of severe perniciousness of malaria itself and lack of self-protective mentality and measures as well as wrong practice to treat malaria as common cold or fever empirically by malaria patients [20]. On the other hand, it is because of widespread misdiagnosis and malpractice on malaria patients in primary healthcare institutions [17, 18]. The robust multiple healthcare system in China once helped reduce indigenous malaria cases efficiently, but presently, it struggles to manage infrequent imported cases. It may need some targeted strategies to cope with the challenge of imported malaria. This study was designed to investigate the possible relationship between imported malaria cases and healthcare institutions based on imported malaria cases in Henan Province during 2012–2017 with a specific focus on the critical problem in imported malaria, as a precursor to targeted strategies on management of imported malaria cases.

## Methods

### Study design and data collection

A retrospective study was conducted to explore the diagnostic and clinical characteristics of imported malaria cases from 1 January, 2012 to 31 December, 2017 in Henan Province using routine surveillance data. All data were from the Centre for Disease Control and Prevention (CDC) of Zhengzhou City, the capital of Henan Province. Data were obtained by combining surveillance data and epidemiological data according to a unique case reporting code. The surveillance data and epidemiological data were from the Infectious Diseases Information Reporting Management System (IDIRMS) and the Parasite Diseases Information Reporting Management System (PDIRMS) respectively. Information on every malaria case was carefully reviewed and verified by checking original paper records, including age, gender, native place, travel history, countries visited, purpose of visits, date of return to China, date of onset, date of first attendance, diagnosis, name of medical institution, date of final diagnosis, specific type of complications. All reported malaria cases during the study period in Henan Province were imported cases.

### *Plasmodium* species confirmation and variable definition

Malaria cases were initially diagnosed at primary healthcare institutions, such as clinic, hospital, and CDC, by means of microscopy, rapid diagnostic test (RDT) or both, and then confirmed by nested polymerase chain reaction (nPCR) and microscopy at the Henan Provincial Reference Laboratory for Malaria Diagnosis.

The ranking of healthcare facilities for initial diagnoses represented the allocated medical resources. The ranks were determined by the name of healthcare institution and the corresponding administrative level, among which provincial was the highest level. Village clinic and private clinic were generally of low quality at least in terms of malaria diagnoses. In addition, village clinic was similar to private clinic in rural China and most developed from the previous ‘barefoot doctors’ who were farmers receiving minimal basic medical and paramedical training and working in rural villages in China. The rank of administrative level in China is Province > municipality > county > township > village, e.g., municipality is subject to Province. Healthcare facilities of higher administrative level tend to have better medical resources.

The results of initial diagnosis were malaria and other diseases, among which malaria was defined as correct diagnosis, while other diseases were defined as misdiagnosed. The time interval was calculated by subtracting the former date from the latter date, which generated inevitably zero as a result. It was notable that zero meant both events happened on the same day. In this study, 1 day was equal to 24 h.

### Statistical analysis and model parameter

Data were checked using Microsoft Excel 2016 by three researchers independently. After calculating and recoding of variables, data were imported into SPSS 23.0 where all descriptive and statistical analyses were done. Categorical variables were described with number and percent and continuous variables were described with percentiles, mean, and 95% CI. Chi-square test was used to compare the differences of categorical variables. Normality test was conducted to continuous variables and then Wilcoxon Rank-Sum test was carried out. A two-sided P value less than 0.05 was considered statistically significant.

Among the four decision tree methods available in SPSS, Chi-squared Automatic Interaction Detection (CHAID) and Classification and Regression Tree (CRT) stood out for highest accuracy and epidemiological plausibility of the structure. A reasonable strategy to construct the tree was adopted to get the optimal tree model [21–27]. Firstly, potential variables related to dependent variable in terms of temporal sequence, logic, and profession were selected out, all of which were set as independent variables to generate the tree as large as possible. Secondly, both outstanding variables in the tree and significant variables in the table of importance to model of independent variables were chosen to attempt the most concise and accurate tree. Thirdly, accompanied by different method and parameter adjustment, various variables combinations were tried. Parameters of the final CHAID tree model were set as follows: maximum tree depth as 4, minimum number of cases in parent node as 20 and in child node as 10, significance level of CHAID as 0.05. Those of the final CRT were as follows: importance to model of independent variables, minimum number of cases in parent node as 10 and in child node as 5. In addition to those stated above, others were default settings.

## Results

Among the 371 imported malaria cases of this study, males were the majority, 366 (98.7%), and the median of age was 37 years, with a range of 17 to 67 years. There were no deaths in the sample during the study period. The malaria cases were mostly from *P. falciparum*: 319 (86.0%), and cases due to *Plasmodium ovale*, *P. vivax* and *Plasmodium malariae* were 34 (9.2%), 13 (3.5%) and 5 (1.3%), respectively (Table 1).

**Table 1:** General and medically related characteristics of imported malaria cases

Characteristics (n = 371)	Number (%)
Gender	
Female	5 (1.3)
Male	366 (98.7)
Age (years)	
Median (IQR)	37 (30, 46)
Malaria parasite type of patient infection	
*Plasmodium falciparum*	319 (86.0)
*Plasmodium ovale*	34 (9.2)
*Plasmodium vivax*	13 (3.5)
*Plasmodium malariae*	5 (1.3)
Rank of healthcare facilities for the initial diagnosis	
Provincial healthcare institutions	120 (32.4)
Municipal healthcare institutions	74 (19.9)
County healthcare institutions	59 (15.9)
Township health centre	17 (4.6)
Village clinic	46 (12.4)
Private clinic	55 (14.8)
Initial diagnosis	
Misdiagnosis	189 (50.9)
Correct diagnosis	182 (49.1)
Complications developed	
Yes	117 (31.5)
No	254 (68.5)
Drug therapy	
Artemisinin	351 (94.6)
Quinine	1 (0.3)
Unknown	19 (5.1)

Days	P_10_, P_25_, P_50_, P_75_, P_90_
Days of taking medicine (n = 320)	4, 5, 7, 8, 9
Time interval between onset and initial diagnosis	0, 0, 1, 2, 5
Time interval between initial diagnosis and final diagnosis	0, 1, 2, 5, 8.8
Time interval between onset and final diagnosis	1, 2, 4, 7, 12.8
Time interval between onset and drug therapy	0, 1, 3, 6, 12

### Access to healthcare and consequences

The top three healthcare institutions attended for first visit by malaria cases were provincial: 120 (32.3%), municipal: 74 (19.9%), and county: 59 (15.9%); the minimum presented to township health centre: 17 (4.6%). The time interval between onset and initial diagnosis of malaria cases in those three healthcare institutions was longer than other healthcare institutions (Table 2). More than half of malaria cases were misdiagnosed on their first visit and concerning complications affected 31.5%. The concerning complications included severe liver/kidney/brain lesions, severe anaemia, haemolysis, shock, septicaemia, coma, and pulmonary oedema. More than three-quarters of the malaria patients were present at healthcare institutions for the first time within 72 h of their onset. Final diagnosis test of most authority and accuracy, the confirmation test, was conducted for about half of the patients within 72 h after initial diagnosis since multilevel detection and confirmation were obligatory for malaria, a notifiable disease in China. What paralleled with above was that the majority of malaria cases received anti-malarial therapy almost at the same time as final diagnosis test. About 95% of patients were treated with artemisinin: for around 7 days, which often included artemisinin for 2–3 days and quinine for the remaining days (Table 1).

**Table 2:** Time interval between onset and initial diagnosis of malaria cases in multiple healthcare institutions

Healthcare institutions	Time interval between onset and initial diagnosis Mean (95% CI)
Provincial	3.48 (1.97–4.98)
Municipal	1.99 (1.16–2.81)
County	5.14 (2.14–8.14)
Township health centre	0.56 (0.09–1.04)
Village clinic	1.15 (− 0.25 to 2.55)
Private clinic	1.15 (0.34–1.95)

### Factors influencing initial diagnosis and malaria complications

Some 50.9% malaria patients were initially misdiagnosed. There was no significant difference in both age and malaria parasite between initial diagnosis groups. The healthcare institutions that malaria patients visited first misdiagnosed patients as follows: 15 of 120 patients at provincial, 113 of 118 patients in township health centre, village and private clinics, and 61 of 133 patients in municipal and county. The misdiagnosed patients had their first visit to healthcare institutions and got their initial diagnosis within significantly fewer days and had significantly more days taking medicine (Table 3).

**Table 3:** Influential factors of the malaria cases at initial diagnosis

Influential factors	Initial diagnosis (% or 95% CI)		P value
	Misdiagnosis	Correct diagnosis	
Gender			
Female	2 (40)	3 (60)	0.966
Male	187 (51.1)	179 (48.9)	
Age (years)	38.21 (36.82–39.59)	37.72 (36.25–39.19)	0.431
Malaria parasite type of patient infection			
*Plasmodium falciparum*	163 (51.1)	156 (48.9)	0.123
*Plasmodium ovale*	16 (47.1)	18 (52.9)	
*Plasmodium vivax*	5 (38.5)	8 (61.5)	
*Plasmodium malariae*	5 (100)	0 (0)	
Rank of healthcare facilities for the initial diagnosis			
Provincial healthcare institutions	15 (12.5)	105 (87.5)	< 0.001
Municipal healthcare institutions	29 (39.2)	45 (60.8)	
County healthcare institutions	32 (54.2)	27 (45.8)	
Township health centre	14 (82.4)	3 (17.6)	
Village clinic	45 (97.8)	1 (2.2)	
Private clinic	54 (98.2)	1 (1.8)	
Time interval between onset and initial diagnosis	2.01 (1.14–2.88)	4.87 (1.7–8.05)	< 0.001
Days of taking medicine	7.25 (6.83–7.67)	6.22 (5.9–6.55)	< 0.001

Most complications affected patients infected with *P. falciparum*; fewest complications affected patients infected with *P. malariae*. Relatively fewer complications occurred in patients attending healthcare institutions of higher administrative level for their first visit and in patients correctly diagnosed initially. There were 20.8, 31.1 and 27.1% complications in patients initially visiting provincial, municipal, and county healthcare institutions, respectively. In contrast, there were 47.1, 21.7 and 63.6% complications in patients initially visiting township health centre, village clinic, and private clinic, respectively. While the proportion of complications in patients with correct initial diagnosis was 18.1%, it was 44.4% in patients with incorrect initial diagnosis. Furthermore, malaria patients with complications tended to have significantly shorter time interval between onset and initial diagnosis, and time interval between onset and drug therapy, however, significantly longer time interval between initial diagnosis and final diagnosis as well as more days of taking medicine (Table 4).

**Table 4:** Influential factors for complications developed among the malaria cases

Influential factors	Complications developed (% or 95% CI)		P value
	Yes	No	
Gender			
Female	2 (40)	3 (60)	1
Male	115 (31.4)	251 (68.6)	
Age (years)	38.97 (37.29–40.64)	37.51 (36.26–38.76)	0.096
Malaria parasite type of patient infection			
*Plasmodium falciparum*	109 (34.2)	210 (65.8)	0.034
*Plasmodium ovale*	7 (20.6)	27 (79.4)	
*Plasmodium vivax*	1 (7.7)	12 (92.3)	
*Plasmodium malariae*	0 (0)	5 (100)	
Rank of healthcare facilities for the initial diagnosis			
Provincial healthcare institutions	25 (20.8)	95 (79.2)	< 0.001
Municipal healthcare institutions	23 (31.1)	51 (68.9)	
County healthcare institutions	16 (27.1)	43 (72.9)	
Township health centre	8 (47.1)	9 (52.9)	
Village clinic	10 (21.7)	36 (78.3)	
Private clinic	35 (63.6)	20 (36.4)	
Initial diagnosis			
Misdiagnosis	84 (44.4)	105 (55.6)	< 0.001
Correct diagnosis	33 (18.1)	149 (81.9)	
Time interval between onset and initial diagnosis	1.62 (1.02–2.21)	4.24 (1.90–6.59)	0.052
Time interval between initial diagnosis and final diagnosis	5.16 (4.08–6.25)	3.17 (2.66–3.69)	< 0.001
Time interval between onset and final diagnosis	6.78 (5.57–7.99)	7.42 (5.05–9.78)	0.002
Time interval between onset and drug therapy	5.69 (4.60–6.79)	6.53 (4.16–8.90)	0.001
Days of taking medicine	7.71 (7.16–8.25)	6.26 (5.97–6.54)	< 0.001

### Decision tree model

**Fig. 1:**
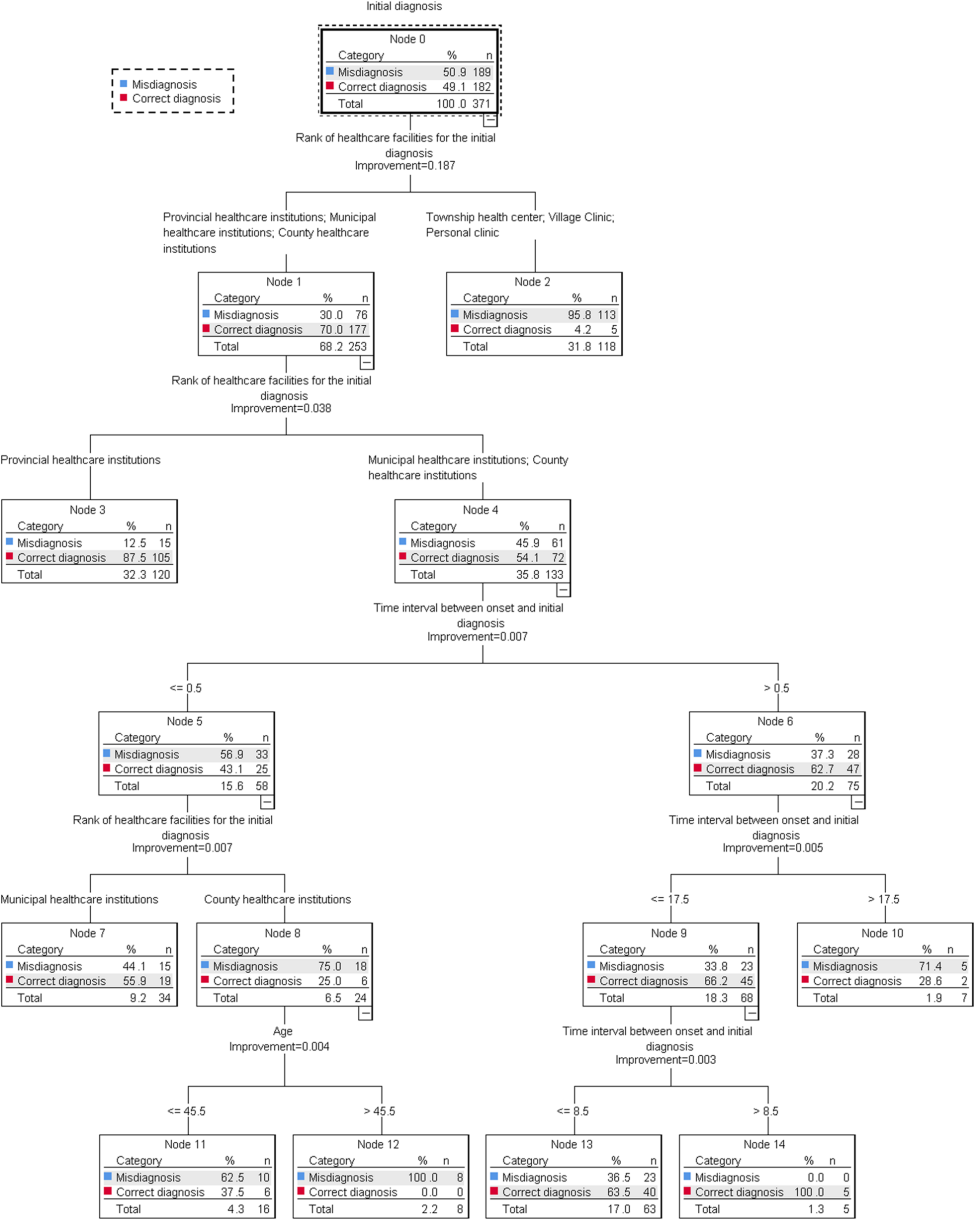
Tree model of initial diagnosis as dependent variable by CRT method

Diverse organizations of variables, growing methods as well as parameter settings of tree model were tested to find the relatively concise decision tree model with optimal accuracy and epidemiological plausibility. When initial diagnosis was taken as dependent variable, three independent variables were found to help build the optimal model by means of CRT, which were rank of healthcare facilities for the initial diagnosis, time interval between onset and initial diagnosis, and age respectively (Fig. 1). The nodes of this tree model reconfirmed some knowledge claimed above. One of them was by node 2, 3, 4, 7, and 8. There was an increasing percentage of initially misdiagnosed patients in provincial, municipal and county healthcare institutions, and the remainder of healthcare institutions as a whole, successively. Another one was by node 5, 13, 14, and 10. When the days between onset and initial diagnosis were < 0.5, 0.5–8.5 and 8.5–17.5, the percentage of initially misdiagnosed patients decreased from 56.9 to 36.5%, with 0 as an end, and then increased to 71.4% after the boundary of 17.5. On the whole, data consisting of 371 cases were made good use in this model where it had a relatively high classification accuracy of 82.2%. Rank of healthcare facilities for the initial diagnosis as independent variable was of most importance to the tree, which meant it was the most significant factor influencing whether malaria patients were correctly diagnosed or not at their first visit.

**Fig. 2:**
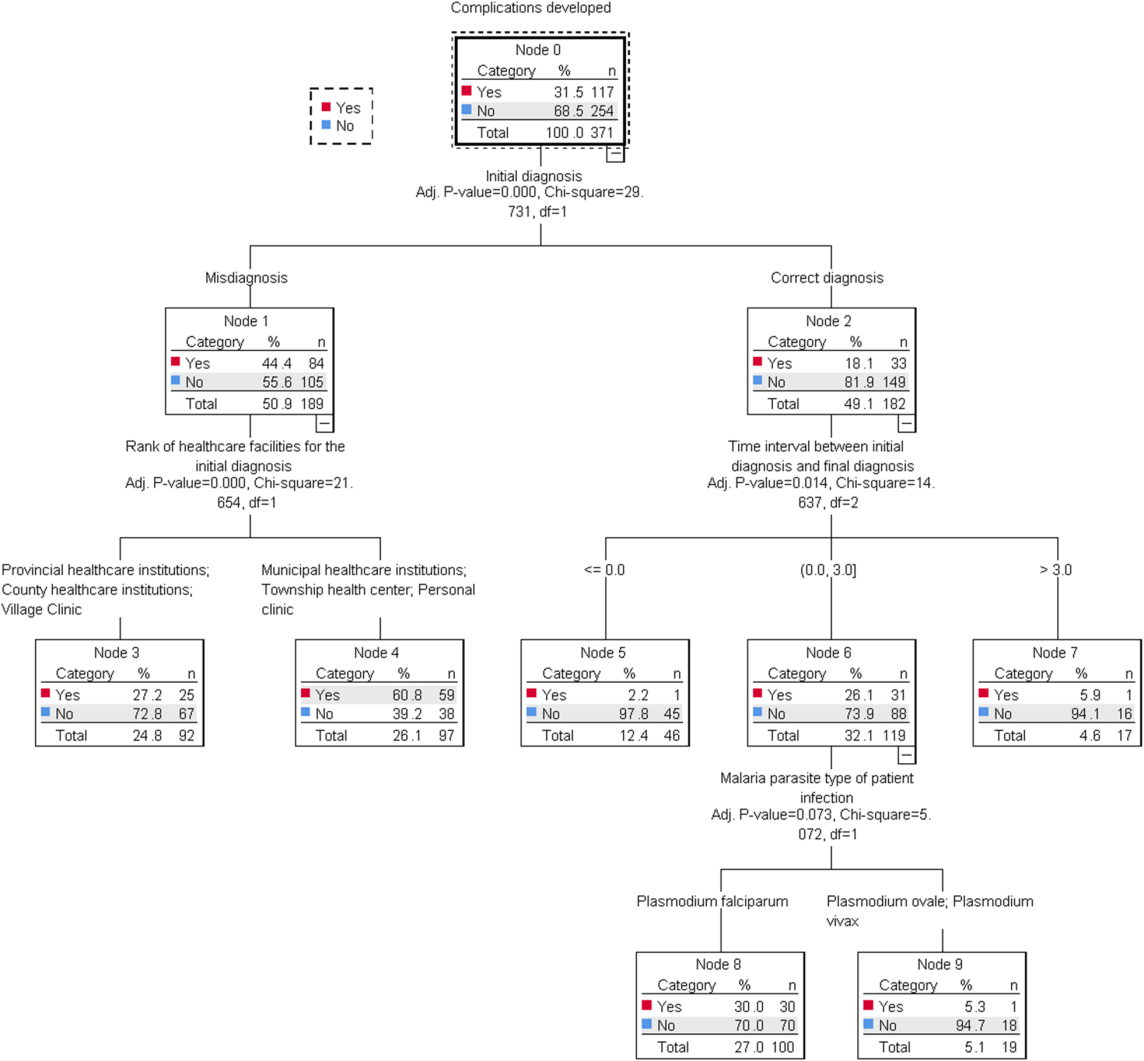
Tree model of complications developed as dependent variable by CHAID method

In the second decision tree model using CHAID method, complications developed as the dependent variable, the four independent variables included were initial diagnosis (P < 0.001), rank of healthcare facilities for the initial diagnosis (P < 0.001), time interval between initial diagnosis and final diagnosis (P = 0.014), and malaria parasite type of patient infection (P = 0.073) (Fig. 2). Initial diagnosis, the dependent variable of first tree model, became the most prominent factor underlying complications developed in the second tree model. Apparently, there were significantly more complications in malaria patients misdiagnosed initially compared with patients correctly diagnosed initially. Cases infected by *P. falciparum* contributed much more to complication frequency than cases infected by *P. ovale* and *P. vivax*.

## Discussion

In the present study, the cases of malaria were imported from other countries where they had worked mainly as labourers and entrepreneurs; few cases were female and the majority of this sample were middle-aged and young [8]. *Plasmodium falciparum* is the most common species in African countries from where most malaria cases reported in Henan Province were imported. Patients infected by *P. falciparum* were more likely to be misdiagnosed and to develop complications in this study. *Plasmodium falciparum* is the most lethal form of parasite of human malaria, which develops rapidly with extreme and complex symptoms, sometimes followed by systemic complications that often result in death [28, 29].

When people with malaria return home from the countries where they were infected they normally plan a visit to certain healthcare facilities for treatment at their convenience, bearing in mind that there are always better medical services in healthcare institutions of higher administrative level in China. In advanced economies superior medical resources appear to be embedded. Because of the existence of marketing, China’s economy has always grown much stronger in areas of a higher administrative level. In contrast, there is disparity in the availability of health services and significant gaps in awareness of malaria and affordability by inhabitants at different administrative levels to the extent of severity of the illness in malaria patients [16, 30]. Therefore, for a number of malaria cases, provincial and municipal healthcare institutions served as their first option because many of them were near cities on their way home, or they lived in cities where better medical care gave them more trust and security. Among those cases living away from cities, in rural areas, they were inclined to frequent local healthcare institutions nearby. In rural areas, patients usually have a long way to go to county healthcare institutions. They treat their unexplained fevers as cold and take empirical medicine instead of visiting distant healthcare facilities, mostly because they are unaware of malaria [29]. County healthcare institutions are of higher profession and authority while village and private clinics are closest in rural areas, so the number of malaria cases initially diagnosed in these healthcare institutions were about the same as present sample. It is proven from the results that patients visiting private clinic, village clinic and township health centre had the shortest period from onset to initial diagnosis, while patients visiting county healthcare facilities had the longest period at first visit. The county healthcare institution of this study was relatively the least accessible but the most reliable healthcare institution in rural areas. It should be noted that the fewest patients were present at the township health centre with the shortest time interval because they maybe happened to live near the township.

It is noted that there are fewer misdiagnosed cases and complications formed at initial diagnosis in healthcare institutions of higher administrative level and that significantly more complications arose in malaria patients who are initially misdiagnosed. This may mean that misdiagnoses arising from poor performance in malaria diagnosis by healthcare institutions contributes to delays in treatment, which eventually leads to complications [31].

For initially misdiagnosed cases of malaria and patients with complications, a shorter time between onset and initial diagnosis was observed. On the one hand, most initially misdiagnosed patients appeared for initial diagnosis in healthcare institutions whose level of administration was lower than municipal level, where misdiagnosis of malaria was very common. In other words, the majority of patients who were initially misdiagnosed were from rural areas where medical services were worse and patients were used to visiting closer healthcare institutions so that the time interval between onset and initial diagnosis was shorter [32]. On the other hand, patients with severe malaria tended to visit healthcare institutions in a shorter time and they were more likely to be misdiagnosed. Cases with rapid onset as well as severe and complicated symptoms will seek medical help in shorter time. That means they have less time to visit a doctor but are more likely to be misdiagnosed and to develop complications. At the same time, as complications arise, it will be more difficult to diagnose patients correctly.

The decision tree model reinforces what was found and expresses it. Decision tree model of initial diagnosis defined township health centre, village clinic and private clinic as homogenous, with the largest proportion of initial misdiagnosis. It also listed municipal healthcare institutions and county healthcare institutions for initial diagnosis with a reasonable proportion of misdiagnosis. This suggests further that these healthcare institutions have similar capacity for malaria diagnosis. In the second decision tree model, dependent variable of complications developed, initial diagnosis became the most outstanding independent variable, which shows that initial diagnosis is significantly correlated with complications developed in malaria patients. Among those diagnosed correctly at the first visit, only one complication occurred in malaria cases receiving a final diagnosis within 24 h (node 5), while the remaining complications occurred in malaria cases that received the final diagnosis after longer periods (node 6). This may have shown that the final diagnosis and parasite confirmation in malaria cases with complications, relative to those without complications, would take more time. In other words, complications can make diagnosing and verifying the parasite correctly in patients with malaria more difficult. Besides, cases infected by *P. falciparum* accounted for most complications in malaria cases of node 6, suggesting a correlation between *P. falciparum* infection and complication in malaria patients.

International labourers from Chinese rural areas are the bulk of imported cases of malaria in China [33]. In imported malaria cases an extraordinary level of misdiagnosis and severe complications are observed. Poor performance in awareness, attitudes and practices of international labourers from rural areas, low capacity for malaria diagnosis in lower-level administrative healthcare institutions, and infrequency of malaria cases make these challenging characteristics of imported malaria in China [16, 20, 30]. A critical problem has been observed after a literature review of the challenge to tackle imported malaria in China [30, 34–40]. It is well known that China’s strong healthcare system has wiped out a severe local malaria epidemic through mass intervention and specific strategies, which defines superiority in medical resources of China [41–45]. Nevertheless, the system currently does not seem to be able to handle imported malaria effectively. This is because of the inaccessibility between unusual cases of imported malaria and advanced medical resources. To be exact, imported malaria cases have little knowledge about the association of their febrile symptoms with malaria. Meanwhile, healthcare institutions with advanced medical resources do not have capability to deal with countless febrile patients in remote rural areas. In this case, a clear travel history to malaria-endemic countries of a febrile patient is beneficial but often not available for some hidden causes [46]. There is a call for a definite link between imported malaria cases and healthcare institutions. There is firstly a proposed innovative internet-based system to make this connection a reality. A theoretical framework system recommended to the malaria department of the Ministry of Health in China consists of two sections. One is the database in the charge of Chinese multiple health departments to process fever information in the migrant population. In this way, febrile patients can be sorted to diagnose and treat any case of imported malaria. Another section is the request for every migrant’s mobile app in a way that, e.g., through WeChat which is the most prevalent social software, every migrant person could receive malaria-related information and communicate with corresponding healthcare centres on any febrile symptoms. This is per the law to eliminate malaria in the future. Specifically speaking, Chinese malaria department will launch a network system that combines communication tools or social software to register and manage migrant populations, with more emphasis on Chinese international labourers returning from Africa and Southeast Asia. In the network system, multiple healthcare institutions will keep in touch with their local migrant population. Any febrile cases must get in touch to report their symptoms to healthcare staff without any hesitation. Effective malaria diagnostic tests of high accuracy and drugs for therapy must be accessible in a short time, providing the means of transporting medical services to migrant febrile patients or sending patients to superior healthcare centres [30]. A panel on malaria diagnostic and treatment of high mobility and the combination of microscopy and RDT for diagnosis are recommended here [47]. To ensure the least burden caused by imported malaria, related laws and regulations will become a fact in the future. Any unintended adverse effects arising from incorrect or overdue practice by individuals and healthcare institutions must be detected. Daily data services to improve health literacy, imperative demands to high-risk population, and reliable and productive professional support could be effectively implemented within the system [30, 47, 48].

The pathway to controlling imported malaria and minimizing deaths is by linking malaria-related medical services to the limited migrant febrile population. Therefore, the proposed system would target a small population. Within the system, high quality malaria medical resources could be used to cope with a small number of migrant febrile cases intensively so its effectiveness could be unparalleled. It is an active, rather than passive, imported malaria management system [40] in which each migrant febrile patient communicates closely with and is in the charge of a local healthcare institution. More importantly, there should be a consensus that all Chinese citizens should undertake due obligation to reduce the burden of malaria and spare no effort to help achieve the goal of malaria elimination together with the Chinese Government, particularly in the context of imported malaria.

### Strengths and limitations of the study

This study of imported malaria cases interacting with healthcare institutions in Henan Province proposed a web-based system combined with WeChat aimed at imported malaria. There are some limitations in the present study. If the time of malaria onset and initial diagnosis in IDIRMS and PDIRMS could be accurate to the hour, some results would be clearer. The lack of times of visiting healthcare institutions by every malaria case before the final diagnosis and the time for the appearance of complications prevented a more convincing demonstration and causality in the present study. Furthermore, it would be more convincing to apply decision tree analysis for a bigger sample size. In fact, there were fewer than 10 deaths from malaria between 2012 and 2017 in Henan Province, but they did not appear in this sample because the cases in present study received anti-malarial treatment in a superior designated hospital for infectious diseases in Zhengzhou City after malaria confirmation. This may bring about limited bias to this study. The limited sample size in the present study appeals to nationwide researchers to carry out larger studies in broader areas. The proposed system is merely a conceptual framework, in spite of covering the core issue. It still lacks innovative professional content in detail, repeated trials, and assessment and improvement by leading experts.

## Conclusions

Inadequate seeking for medical care by imported malaria patients, and insufficient capacity to diagnose malaria by healthcare institutions of lower administrative level were identified as the major factors associated with complications of imported malaria cases in Henan Province, on which the crucial challenge of imported malaria is based. A web-based system combined with WeChat aimed at imported malaria patients was proposed that would possibly become a stimulation to alleviate the burden of imported malaria.

## Data Availability

The datasets used and analysed during the current study are available from the corresponding author on reasonable request.

## Abbreviations

NMEAP: National Malaria Elimination Action Plan
CDC: Centre for Disease Control and Prevention
IDIRMS: Infectious Diseases Information Reporting Management System
PDIRMS: Parasite Diseases Information Reporting Management System
RDT: rapid diagnostic tests
nPCR: nested polymerase chain reaction
CHAID: Chi-squared Automatic Interaction Detection
CRT: Classification and Regression Tree
